# Development of HER2-specific chimeric antigen receptor T cells for the treatment of breast-to-brain metastasis

**DOI:** 10.1186/2051-1426-3-S2-P121

**Published:** 2015-11-04

**Authors:** Saul Priceman, Brenda Aguilar, Renate Starr, Dileshni Tilakawardane, Anthony Park, Ethan Gerdts, Wen-Chung Chang, Sarah Wright, Stephen J Forman, Christine E Brown

**Affiliations:** 1 City of Hope National Medical Center, Duarte, CA, USA; 2 Beckman Research Institute, City of Hope National Medical Center, Duarte, CA, USA

## 

Adoptive transfer of chimeric antigen receptor (CAR)-engineered T cells has demonstrated robust and durable clinical efficacy in patients with CD19+ B cell malignancies. Broader application of this approach to brain and other advanced solid tumors is an immediate goal for the field and is presently under intense investigation. In 2014, 40,000 individuals in the U.S. alone succumbed to breast cancer, primarily as a result of metastatic disease. Approximately 30 percent of breast cancer patients carry an amplification of the HER2 gene and/or HER2 over-expression, which confers a particularly poor prognosis. Among the most common sites of HER2-positive breast cancer metastases is the brain. For patients with breast cancer that has metastasized to the brain, the 1-year survival rate is a dismal 20 percent. Despite clinical successes in both preventing relapse and treating systemic disease with HER2-targeted therapies, the currently available agents are only modestly effective in managing brain metastasis. Our group has demonstrated safety and transient anti-tumor responses in two U.S. FDA-authorized Phase I clinical trials evaluating local intracranial adoptive transfer of CAR T cells in glioma patients. Our current project builds on this clinical experience with locally administered CAR T cells that specifically target HER2 for the treatment of breast-to-brain metastasis.

Our lab has initiated design and testing of CAR T cell therapy targeting HER2 based on an scFv derived from trastuzumab. We anticipate that local intracranial delivery will enhance therapeutic response, while reducing the likelihood of off-tumor systemic toxicities as previously observed. Importantly, HER2 expression in normal brain tissue is limited, supporting HER2 as a therapeutic target in brain metastasis. Our innovative CAR T cell platform focuses on engineering central memory T cells (Tcm) for therapeutic application, with the intent of improving persistence of T cells after infusion, a critical parameter correlated with ultimate therapeutic success. Herein, we have evaluated several HER2-CAR constructs incorporating either the CD28 or 4-1BB co-stimulatory domain using both *in vitro* T cell functional assays as well as orthotopic patient-derived xenograft models of breast-to-brain metastasis. While HER2-28z and HER2-BBz CAR T cells similarly kill HER2+ breast cancer cells *in vitro*, BBz CARs demonstrate lower induction of the exhaustion marker PD-1 and proliferate better compare with 28z CARs. Both HER2-CARs similarly lead to tumor eradication and prolonged survival. Based on these data, we have successfully developed HER2-specific CAR T cells, and plan to clinically develop these CARs for the treatment of HER2+ metastatic disease.

